# Organic/inorganic phosphorus partition and transformation in long-term paddy cultivation in the Pearl River Delta, China

**DOI:** 10.1038/s41598-023-38369-2

**Published:** 2023-07-10

**Authors:** Xian Tang, Hongyi Liu, Hailong Qin, Jianrong Zhao, Hong Wang, Bo Li, Ying Lu

**Affiliations:** 1 College of Natural Resources and Environment, South China Agricultural University, Guangzhou, 510642 People’s Republic of China; 2 College of Natural Resources and Environment, Anhui Science and Technology University, Chuzhou, 233100 People’s Republic of China

**Keywords:** Environmental sciences, Solid Earth sciences

## Abstract

Identification and quantification of different soil phosphorus (P) fractions level are important for improving agricultural productivity and developing sustainable management practices in these agricultural soils under long-term cultivation. However, few studies have been conducted to investigate P fractions level and their transformation in these soils. This study was conducted to characterize P fractions as affected by different paddy cultivation ages (200, 400-yr and 900-yr) among soils of the Pearl River Delta Plain in China. A sequential chemical fractionation scheme and ^31^P nuclear magnetic resonance spectroscopy (^31^P NMR) were employed to quantify various P fractions and speciation. Results showed soil easily-labile P, moderately-labile P and non-labile P had a positive relationship with total P (TP) and available P (AP). Analysis with ^31^P NMR spectroscopy revealed that inorganic P including orthophosphate (Ortho-P) and pyrophosphate (Pyro-P) increased with cultivation age, while organic species monoester phosphate (Mono-P) and diester phosphate (Diester-P) decreased. Moreover, acid phosphatase (AcP), neutral phosphatase (NeP), exchangeable Ca and sand contents are the main factors that affected the transformation of soil P composition, and non-labile P (Dil.HCl-Pi) and Pyro-P had significant contribution to soil P availability by affecting P activation coefficient. Therefore, long-term paddy cultivation, influenced by these soil parameters including NeP, AcP, exchangeable Ca and sand, accelerated the transformation of soil organic P/non-labile P to inorganic P.

## Introduction

Phosphorus (P) is one of the major nutrients limiting plant growth and agricultural production^1–3^. Transformation between organic and inorganic forms during ecosystem development exerts a crucial influence on soil fertility and ecosystem properties^4,5^. Processes involved in P cycling includes dissolution–precipitation reactions, sorption–desorption interactions between the solution and solid phases, and mineralization-immobilization reactions between organic and inorganic, soluble forms^6^. Organic P constitutes 20–80% of the total P in surface soils and is not directly available to plants. The P of organic form can be converted to soil solution P form, becoming plant-available via biotic processes such as mineralization process^7,8^. However, most solution P transforms to insoluble and tightly bound inorganic phosphates forms and thus become unavailable for plant growth. Thus, the transformation of solution P forms, as well as low native P in soils, make this nutrient limiting for agricultural production in south China.

In contrast, heavily fertilized paddy soils typically have P buildup to levels that far exceed the agronomic optimum required for satisfactory crop production^9^. This excess P may lead to elevated P loss to water bodies and cause eutrophication of lakes, rivers and estuaries^10^. Thus, research on quantifying P fractions as well as the chemical reactions that govern P availability on these paddy soils with long cultivation history is essential to improve our understanding of P requirements for crop production, appropriate management practices, as well as pathways of P transformation and transport in the agroecosystem.

The effect of paddy cultivation on soil P composition has been widely studied among different agricultural soils^9,11^. For example, short-term cultivation has been found to reduce the content of NaHCO_3_-extractable inorganic P (NaHCO_3_-Pi), NaOH-extractable inorganic P (NaOH-Pi), HCl-extractable inorganic P (HCl-Pi) and Residual-P in double-cropping rice system^12^. Huang et al.^9^ found that total P and various P fractions (such as calcium phosphates, organic phosphates, and non-occluded and occluded P) can accumulate to a maximum after 50-yr and 150-yr of cultivation that includes long-term P addition. In addition, soil phosphatase enzymes, including acid phosphatase and neutral phosphatase, play critical roles in organic and condensed P hydrolysis and P availability to crops. The activity of these enzymes can be affected by tillage^13^. Land use and management practices (i.e. tillage, fertilization, and residue input) can significantly increase different fractions of P and phosphatase activities due to the enhanced P binding resulting from an increase in the contact between solution P and soil particles^9,13^. However, most previous researches were based on short-term soil analysis, and little was known about how soil P fractions and speciation responds to crop cultivation over long-term agricultural land use timescales.

Sequential fractionation methodology is commonly used to determine soil forms of elemental constituents. For example, it has been used for determining existing pools of trace metals in contaminated soils^14^. This approach has proven to be effective in understanding of soil P status in previous studies^3,15^ and the chemical sequential fractionation method is recognized as a systematic, comprehensive classification method by soil scholars^16^. The power of this method for P acquisition is increased by additional use of solution ^31^P nuclear magnetic resonance (^31^P NMR) spectroscopy to quantify organic P fractions^17,18^. Use of both fractionation and this NMR analytical method should prove valuable in studying P transformation in soils that differ in cultivation age.

Climate of the Pearl River Delta (PRD) region in south China is humid and subtropical, with over 2000-yr of cultivation^19^. It is well known that high temperature and precipitation can enhance rock weathering, which supplied essential nutrient P to support high biological cycling^20^. Furthermore, enhanced chemical weathering and hydrological transport as well as biological uptake result in low TP in soil and sediments^20^. Therefore, these processes can significantly reduce TP content in regions with tropical and subtropical climate^20^. Over the past three decades, the PRD has undergone rapid industrialization and urbanization, becoming a major manufacturing hub^21^. Due to the rapid economic development in the PRD region, available land for agriculture is decreasing, increasing the need for a revised approach to farmland management^22^. Adjustments are needed accommodate multiple crops, with coexistence of rice, vegetables, fruit trees and other cash crops^23^. A variety of direct and indirect processes induced by land uses can potentially alter the stock, fractions, and availability of soil P^24^. These land use demands have increased the need for proper use of nutrients to maximize uptake and crop yields, while minimizing P fixation or nutrient loss to water bodies^25^. In addition, the variations in the fractions and availability of soil P under land uses can be indirectly driven through the changes in soil properties, plant diversity and/or richness, and microbial activity^26^. Previous studies have observed the improved nutrient states including P after farmland being abandoned, because of the increased inputs of plant residues, accelerated microbial turnover, and improved soil physical properties, such as bulk density and soil moisture^27^. Therefore, exploring the transformation processes of soil P composition in faemland (e. g. paddy field) is vitally important for agricultural sustainable development in the PRD region.

Maximizing soil P availability in the PRD region by maintaining adequate amounts of soil P through application of inorganic and/or organic P sources is critical for the long-term sustainability of cropping systems^28^. Previous studies have shown that cultivation can significantly affect soil P composition, content and plant availability, and revealed some mechanisms of soil P transformation^29^. Due to its long agricultural history and subsequent changes in land use, the PRD presents an important location to examine effects of long-term cultivation on agricultural soils and determine potential pathways of transformation between soil P forms.

This study was conducted to investigate the impact of time of cultivation in different soil P fractions and species, and whether it influences the abundance and composition of P-cycling-related phosphatase. We hypothesized that (i) time of paddy cultivation would alter amount and distribution of P and its bioavailability; (ii) P composition would differ across soils and with depth in individual locations; and (iii) the abundance and species of P-cycling-related phosphatase enzymes would be closely linked to P composition and other physicochemical factors.

## Results

### Total P and soil P fractions by sequential chemical extraction

Soil total P (TP) ranged from 0.6 to 2.0 g kg^−1^ with an average of 0.79 g kg^−1^ (Table 1). It was highest in the 900-yr soil. Soil AP ranged from 3.9 to 227.2 mg kg^−1^ and was also highest in the 900-yr soil (Table 1). The depth distribution of the P activation coefficient (PAC) was generally uniform throughout soil profiles for same cultivation age (Table 2). The PAC ranged from 0.7 to 3.0% with an average of 1.63%, and was greatest in the 900-yr soil (Table 2). Accumulation of AP in both the subsoils indicated that P addition by paddy cultivation not only resulted in surface P enrichment but also caused significant movement of P to lower horizons.

**Table 1:** Physiochemical characteristics of the studied soils from each soil profile.

Sampling no.	Soil genetic horizon	Depth (cm)	Soil pH	Sand (%)	Silt (%)	Clay (%)	SOC (g kg^−1^)	CEC (cmol kg^−1^)	ECa (cmol kg^−1^)	EMg (cmol kg^−1^)	Fe_c_ (g kg^−1^)	Fe_d_ (g kg^−1^)
PF_200_	Ap1	0–14	6.65	14.7	51.0	34.4	22.4	17.6	18.4	2.82	7.21	45.3
	Ap2	14–23	6.54	13.4	49.5	37.2	21.1	16.5	20.7	1.51	6.93	44.0
	Br1	23–60	6.65	11.7	51.3	37.0	18.7	16.6	21.6	3.25	9.11	46.3
	Br2	60–90	6.73	8.89	52.6	38.5	12.1	14.9	18.4	2.83	29.7	32.1
	G	90–120	7.04	21.7	52.4	25.9	10.0	11.5	45.1	3.46	13.3	26.1
PF_400_	Ap1	0–13	7.02	30.2	41.9	27.9	18.0	15.2	13.6	1.41	6.46	37.6
	Ap2	13–28	6.98	16.1	43.5	40.4	13.6	16.0	21.1	2.70	1.61	43.8
	Br1	28–45	6.89	3.84	49.6	46.5	8.08	17.2	23.1	3.24	2.10	53.4
	Br2	45–73	6.96	3.98	49.6	46.4	7.98	18.4	22.1	6.38	3.59	54.5
	Br3	73–92	6.89	2.62	53.9	43.5	8.70	16.1	32.6	6.89	5.43	49.9
	G	92–118	6.72	3.79	57.9	38.3	11.4	15.5	44.7	5.78	33.8	34.3
PF_900_	Ap1	0–19	6.20	35.8	37.9	26.3	13.7	14.4	11.8	1.58	9.20	29.0
	Ap2	19–32	6.43	30.4	36.3	33.4	9.57	14.4	20.5	1.61	5.19	33.6
	Br1	32–59	6.65	22.3	40.3	37.4	5.92	15.1	33.0	3.28	3.27	38.5
	Br2	59–82	6.75	8.38	50.2	41.4	4.14	12.9	12.7	2.74	1.11	40.7
	G	82–113	6.87	3.90	47.7	48.4	4.73	14.3	12.8	2.91	1.10	36.8

PF_200_, PF_400_ and PF_900_ means paddy field with cultivation ages of 200, 400-yr and 900-yr, respectively. SOC, soil organic C; CEC, cation exchange capacity; ECa, exchangeable calcium; EMg, exchangeable magnesium; Fe_d_, free iron; Fe_c_, active iron.

**Table 2:** The contents distribution of different **s**oil P fractions and PAC under different cultivation ages and soil depths.

Sampling no.	Soil genetic horizon	Depth (cm)	NaHCO_3_-Pi (mg kg^−1^)	NaOH-Pi (mg kg^−1^)	Dil.HCl-Pi (mg kg^−1^)	Conc.HCl-Pi (mg kg^−1^)	Residual-P (mg kg^−1^)	Pi (mg kg^−1^)	NaHCO_3_-Po (mg kg^−1^)	NaOH-Po (mg kg^−1^)	Conc.HCl-Po (mg kg^−1^)	Po (mg kg^−1^)	AP (mg kg^−1^)	TP (g kg^−1^)	PAC (%)
PF_200_	Ap1	0–14	32.7	109	161	301	137	741	15.9	92.2	38.4	146	11.5	0.89	1.30
	Ap2	14–23	27.5	92.2	168	300	131	719	13.3	42.0	29.1	84.4	11.7	0.81	1.44
	Br1	23–60	18.3	51.4	93.2	254	142	559	8.90	26.1	19.5	54.5	4.79	0.59	0.81
	Br2	60–90	28.6	58.9	211	150	106	555	8.58	28.9	21.5	59.0	12.2	0.58	2.10
	G	90–120	32.0	62.8	246	113	97.2	551	2.50	29.9	18.2	50.6	13.3	0.60	2.22
PF_400_	Ap1	0–13	48.1	135	188	266	142	779	17.5	98.4	56.3	72.3	24.6	0.98	2.49
	Ap2	13–28	18.7	59.7	79.3	288	167	613	6.21	22.3	36.0	64.5	7.46	0.70	1.07
	Br1	28–45	11.0	55.9	36.7	303	167	573	5.50	11.1	19.6	36.2	3.90	0.59	0.66
	Br2	45–73	14.8	67.8	77.3	352	159	671	6.74	14.6	19.0	40.4	6.99	0.69	1.02
	Br3	73–92	22.1	77.0	113	327	165	704	5.18	12.3	26.0	43.5	9.49	0.74	1.28
	G	92–118	39.7	75.8	277	164	149	705	2.79	19.1	24.5	46.4	16.8	0.71	2.37
PF_900_	Ap1	0–19	508	494	563	231	132	1929	41.2	105	45.3	191	227	2.04	1.00
	Ap2	19–32	63.8	111	254	271	117	817	36.8	19.0	32.5	88.4	26.8	0.89	3.02
	Br1	32–59	56.0	107	229	352	139	884	5.76	6.47	35.1	47.3	21.6	0.89	2.41
	Br2	59–82	21.9	75.2	31.3	255	138	522	9.02	4.61	16.4	30.0	9.66	0.53	1.81
	G	82–113	10.2	38.5	26.7	155	111	342	2.37	6.31	15.9	24.6	3.67	0.36	1.01

PF_200_, PF_400_ and PF_900_ were the cultivation ages of 200, 400-yr and 900-yr, respectively; Pi, total inorganic P; Po, total organic P; NaHCO3-Pi, NaHCO3-extractable inorganic P; NaHCO3-Po, NaHCO3-extractable organic P; NaOH-Pi, NaOH-extractable inorganic P; NaOH-Po, NaOH-extractable organic P; Dil.HCl-Pi, dilute HCl-extractable inorganic P; Conc.HCl-Pi, concentrated HCl-extractable inorganic P; Conc.HCl-Po, concentrated HCl-extractable organic P; Residual-P, residual inorganic and organic P; AP, available P; PAC, P activation coefficient.

**Figure 1:**
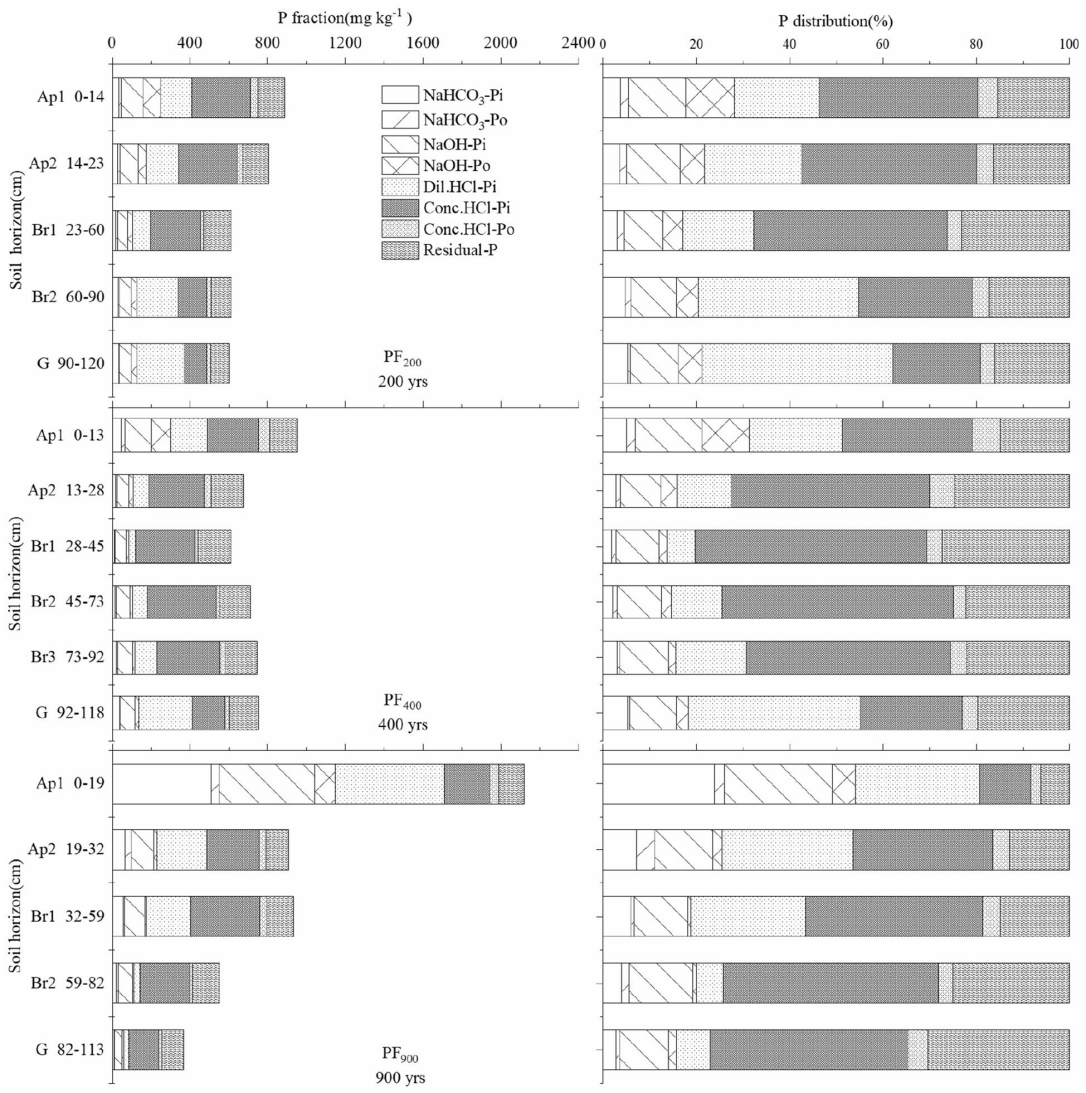
Distribution of sequential fractionation of P in soil profiles across three soil cultivation ages. The depth of a specific soil horizon is not directly comparable to the width of bar. (NaHCO_3_-Pi, NaHCO_3_-extractable inorganic P; NaHCO_3_-Po, NaHCO_3_-extractable organic P; NaOH-Pi, NaOH-extractable inorganic P; NaOH-Po, NaOH-extractable organic P; Dil.HCl-Pi, dilute HCl-extractable inorganic P; Conc.HCl-Pi, concentrated HCl-extractable inorganic P; Conc.HCl-Po, concentrated HCl-extractable organic P; Residual-P, residual inorganic and organic P; PF_200_, PF_400_, and PF_900_ were the cultivation ages of 200, 400-yr and 900-yr, respectively).

Soil P fraction in these alluvial soils was dominated by inorganic P (Pi) the fraction extracted by concentrated HCl (Conc. HCl-Pi) (10.9–49.7% of TP) while NaOH-Po accounts for 0.8–10.4% of TP (Fig. 1). The TP in surface horizons (Ap1) highest in the soil with 900 yrs of cultivation (Table 2, Fig. 1). In the subsoils (below the plow pan), TP decreased across cultivation age (Table 2), which can be mainly attributed to a decrease in the P fraction extracted by 1.0 M HCl (Dil.HCl-Pi) (regarded as Ca-bound P) despite a slight increase in the less readily extractable fractions (such as residue P) (Fig. 1). The distribution of soil P in subsoil was relatively unaltered in the first 400 yrs of cultivation but changed substantially in the older soil (900-yr) that accompanied formation of Fe- and clay-enriched horizons (Fig. 1). For example, TP was generally higher in the Br2 and G horizons of the 200-yr soil and the Br3 and G horizons of 400-yr soil than other subsoils (Fig. 1). The average topsoil Pi and Po concentrations were 0.73 and 0.07 g kg^−1^, respectively, comprising 90.8% and 9.2% of the TP_sum_ (the sum of Pi and Po), respectively. In addition, the labile Po and Pi (NaHCO_3_-Pi and NaHCO_3_-Po) constitutes a small fraction of the total composition, accounting for only 1.8 to 24.0% and 0.6 to 4.1% of the total extractable P reservoir, respectively (Fig. 1, Table 2).

**Figure 2:**
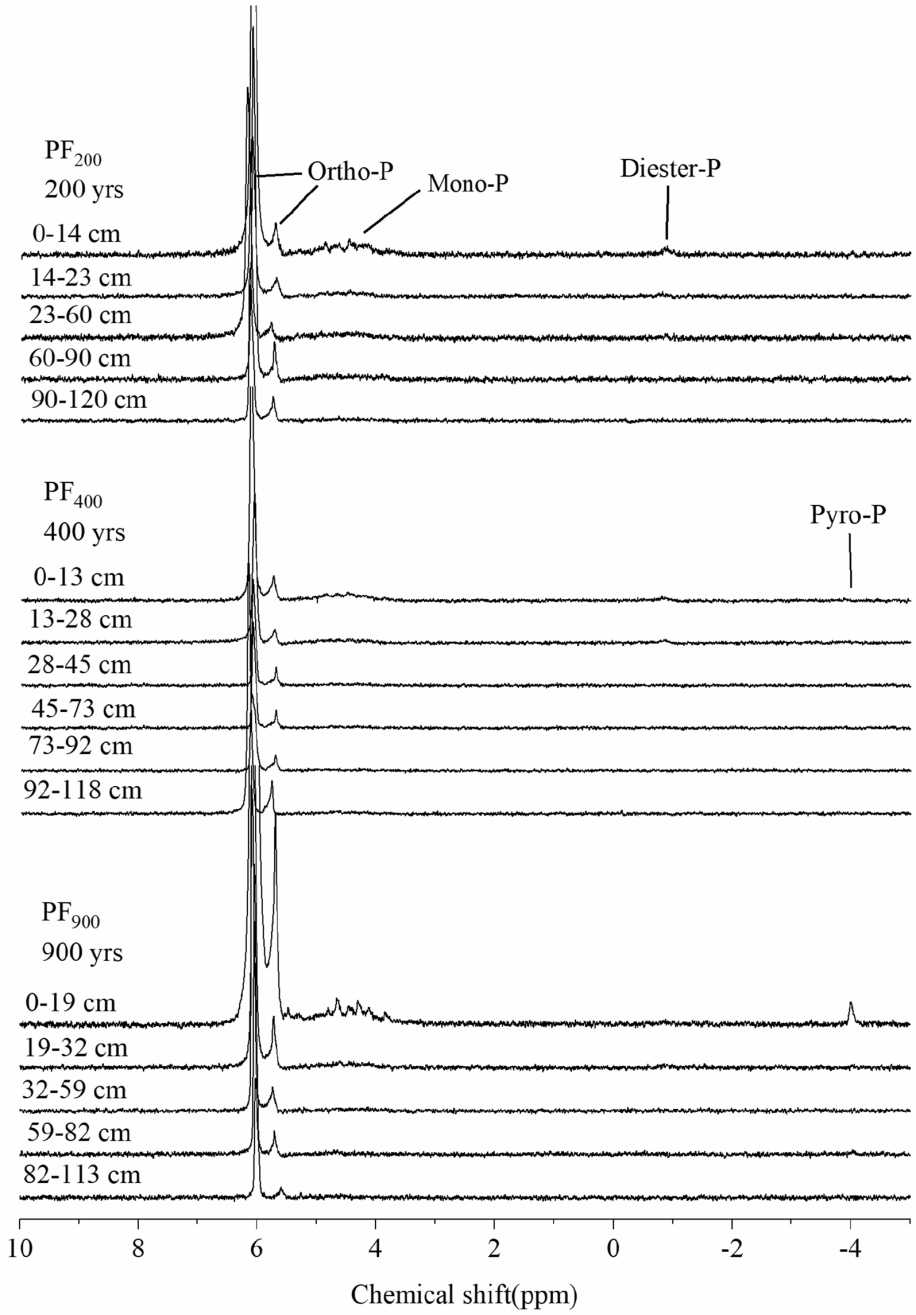
^31^P nuclear magnetic resonance spectra of NaOH-EDTA extracts for selected soils in different chemical shift, showing peaks and corresponding P compounds. (Ortho-P, orthophosphate; Mono-P, monoester phosphate; Diester-P, diester; Pyro-P, pyrophosphate. PF_200_, PF_400_, and PF_900_ were the cultivation ages of 200, 400-yr and 900-yr, respectively).

Soil P pools were significantly affected by cultivation age due to the changes of P fractions. The Pi pools including moderately-labile Pi (NaOH-Pi) and non-labile Pi (Conc.HCl-Pi) at the 20–120 cm depth increased in the 400-yr soil compared to the 200-yr paddy soil (Figs. S2, S3). In contrast, Po pools including easily-labile Po (NaHCO_3_-Po) and moderately-labile Po (NaOH-Po) between 20 and 120 cm showed a decrease in the 400-yr site, The Pi pools (including easily-labile Pi (NaHCO_3_-Pi), moderately-labile Pi (NaOH-Pi) and non-labile Pi (Dil.HCl-Pi) within 0–60 cm for the 900-yr soil showed an increase compared with 400-yr soil. With the exception of NaHCO_3_-Pi and NaHCO_3_-Po, Pi and Po at 60–120 cm tended to decrease progressively with paddy soil development (Figs. S2, S3). Pi and Po pools of moderately-labile P (extracted by 0.1 M NaOH) and non-labile (extracted by 1.0 M HCl, concentrated HCl-Pi and Residual-P) changed in a similar pattern to that of Pi within 60–120 cm (Figs. S2, S3), and changes in these fractions accounted for more than 90% of the changes in TP (Figs. 1, S3). Our results (Figs. 1, 2, S3) suggest that increasing years of cultivation caused redistribution of P forms within soil horizons.

### Soil Pi speciation determined by ^31^P nuclear magnetic resonance (^31^P NMR) spectroscopy

Total P concentration extracted by NaOH-EDTA (TP_alkali_) in paddy soils ranged from 112 to 1543 mg kg^−1^, equivalent to 30.8 to 75.5% of the total P (TP_acid_) in soils (Table 3), respectively. Pi species determined by ^31^P NMR in our study mainly consisted of inorganic orthophosphate (Ortho-P) and pyrophosphate (Pyro-P) (Table 3, Fig. 2). In this study, Ortho-P accounted for 84.1–99.7% of extractable TP_alkali_ and was the major constituent of Pi in all tested soils, while Pyro-P only existed in the Ap horizon of the 400-yr (0.4%) and 900-yr (0.6%) and Br2 horizon of the 900-yr soil. The proportion of Ortho-P increased with cultivation age, ranging from an average of 91.6% to 94.7% in the 200-yr and 900-yr soil profiles, respectively. Moreover, Ortho-P in 200-yr and 400-yr soil was found mostly in G horizon, while primarily existed in the Br horizon in 900-yr soil.

**Table 3:** Relative percentages of P compounds in different chemical shifts as measured by ^31^P nuclear magnetic resonance (^31^P NMR) spectroscopy of NaOH-EDTA extractable total P (TP_alkali_) at each soil layers in three soil profiles.

Sampling no.	Soil genetic horizon	Depth (cm)	TP_alkali_ (mg kg^−1^)	Phosphorus species distribution in different chemical shifts (ppm), % of total P in NaOH-EDTA extract			
				Inorganic ortho-phosphate, 5.0–7.0 ppm	Monoesters, 3.0–5.0 ppm	Diesters, − 2.0 to 0.0 ppm	Inorganic pyro-phosphates, − 5.0 to − 3.0 ppm
PF_200_	Ap1	0–14	300	84.5	14.0	1.5	nd
	Ap2	14–23	285	91.6	8.1	0.3	nd
	Br1	23–60	160	90.2	9.3	0.5	nd
	Br2	60–90	202	93.3	6.7	0.0	nd
	G	90–120	200	98.5	1.5	0.0	nd
PF_400_	Ap1	0–13	239	84.1	15.0	0.5	0.4
	Ap2	13–28	225	91.7	8.1	0.2	nd
	Br1	28–45	138	95.3	4.7	nd	nd
	Br2	45–73	159	97.2	2.8	nd	nd
	Br3	73–92	193	98.0	2.0	nd	nd
	G	92–118	151	99.8	0.2	nd	nd
PF_900_	Ap1	0–19	1543	95.7	3.7	nd	0.6
	Ap2	19–32	296	92.3	6.9	0.9	nd
	Br1	32–59	297	95.2	4.8	nd	nd
	Br2	59–82	193	96.8	2.6	nd	0.6
	G	82–113	112	93.4	6.6	nd	nd

nd, not detected; Moder-P, moderately-labile P; Non-P, non-labile P; Ortho-P, orthophosphate; Mono-P, monoester phosphate; Diester-P, diester; Pyro-P, pyrophosphate; PF_200_, PF_400_, and PF_900_ were the cultivation ages of 200, 400-yr and 900-yr, respectively.

### Soil Po speciation determined by ^31^P nuclear magnetic resonance (^31^P NMR) spectroscopy

Po detected by ^31^P NMR included orthophosphate monoesters (Mono-P) and diesters (Diester-P) (Fig. 2). Mono-P, 1.5–15.0% of NaOH-EDTA extracted P, was higher in 200-yr and 400-yr soils than 900-yr soil (Table 3, Fig. 2). Diester-P were higher in 200-yr soil than in 400- and 900-yr soils, comprising 0.2–1.5% of the NaOH-EDTA extracted P (Table 3). In our study, cultivation age had a large effect on Diester-P (Table 3). The 200-yr soil profile showed higher Diester-P than other soils. Moreover, Mono-P in 200-yr and 400-yr old soils decreased with soil depth, while Mono-P of 900-yr soil increased with depth (Table 3). With increasing of cultivation age, the proportion and composition of soil P compounds changed to some extent. As shown in Fig. 2, Mono-P and Diester-P contents in short-term (200-yr) cultivated soils decreased compared with long-term (900-yr) cultivated soils (Table 3; Fig. 2).

### Soil phosphatase activities

**Figure 3:**
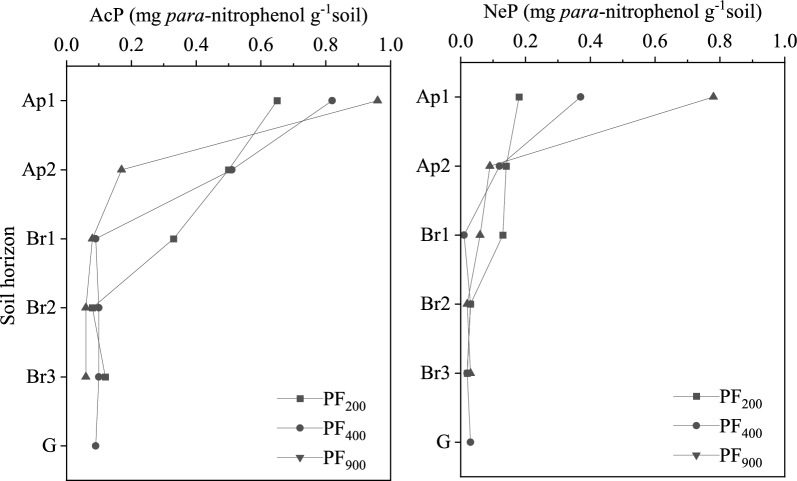
Soil phosphatases activities in three cultivation ages. (AcP, acid phosphatase activities; NeP, neutral phosphatase activities. PF_200_, PF_400_, and PF_900_ were the cultivation ages of 200, 400-yr and 900-yr, respectively).

**Figure 4:**
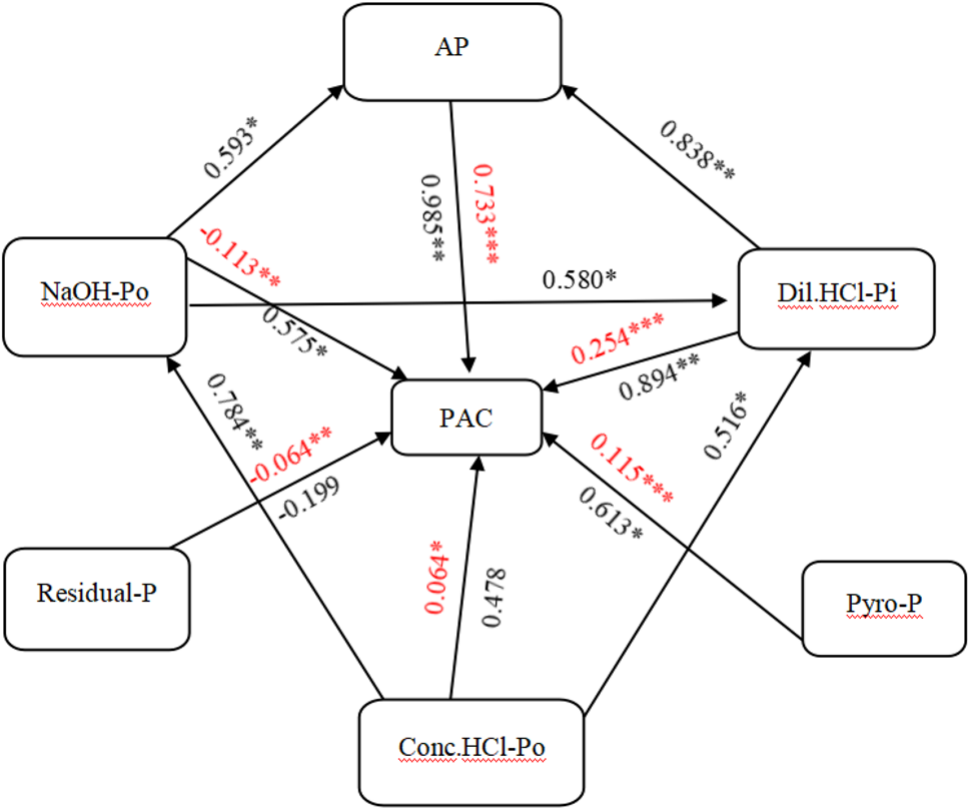
Path analysis between P activation coefficient (PAC), soil physiochemical properties and P composition by sequential chemical extraction and ^31^P nuclear magnetic resonance. N = 16; Red data are direct path coefficients, the black data are correlation coefficients. *, ** and *** indicates an extremely significant correlation at the P < 0.05, P < 0.01 and P < 0.001 level (two-tailed), respectively. (Dil.HCl-Pi, dilute HCl-extractable inorganic P; Conc.HCl-Po, concentrated HCl-extractable organic P; NaOH-Po, NaOH-extractable organic P; Residual-P, residual inorganic and organic P; TP, Total-P; AP, available P; Pyro-P, pyrophosphate).

Acid and neutral phosphatase (AcP and NeP) activities in paddy soils were affected by cultivation age and soil depth (Fig. 4). Overall, AcP activity was greater than that of NeP. The AcP and NeP activities in the Ap1 horizon were higher than that in other horizon, and both activities decreased with depth (Fig. 3). Soil AcP and NeP activities in the Ap1 horizon increased with increasing cultivation age. Paddy soils of 400 years and 900 years in Ap2, Br1 and Br2 horizons had lower AcP and NeP activities compared to that of 200-yr, though similar AcP and NeP activities were found at Br2, Br3 and G horizons for the three soil ages. Thus, long-term cultivation in paddy soils increased AcP and NeP activities at topsoil and upper subsoil (Ap1, Ap2 and Br1) but remained stable in the lower subsoil (Fig. 4).

### The relationships among P compositions, soil properties and phosphatase activities

**Figure 5:**
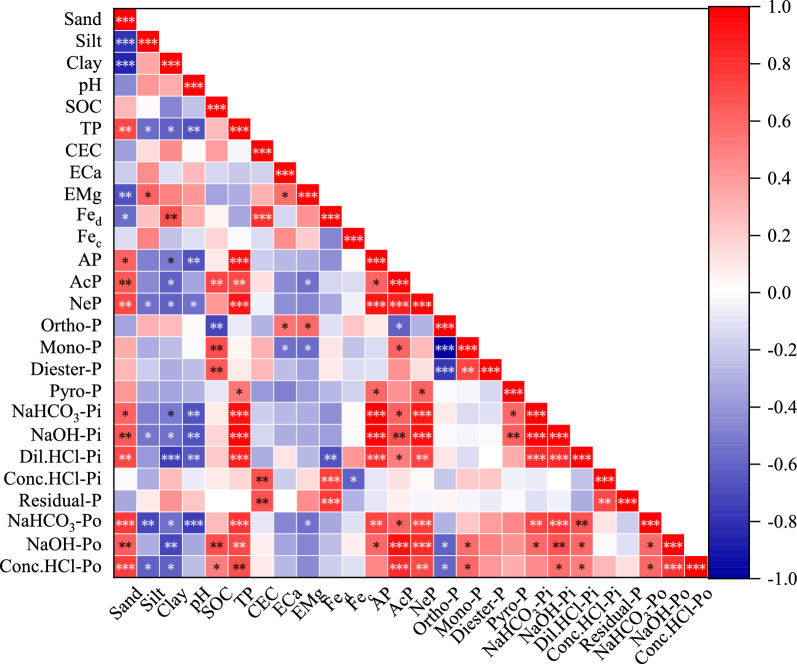
Pearson correlation coefficients using Origin 2021b (OriginLab, New York, USA) between soil physicochemical properties, phosphatases activities and P compositions determined by chemical sequential extraction and ^31^P NMR spectroscopy with different cultivation age and soil depth (*P < 0.05; **P < 0.01, ***P < 0.001). Blue and red denote positive and negative correlations, respectively. (SOC, soil organic C; CEC, cation exchange capacity; ECa, exchangeable calcium; EMg, exchangeable magnesium; Fe_d_, free iron; Fe_c_, active iron; AcP, acid phosphatase activities; NeP, neutral phosphatase activities; Pi, inorganic P; Po, organic P; NaHCO_3_-Pi, NaHCO_3_-extractable inorganic P; NaHCO_3_-Po, NaHCO_3_-extractable organic P; NaOH-Pi, NaOH-extractable inorganic P; NaOH-Po, NaOH-extractable organic P; Dil.HCl-Pi, dilute HCl-extractable inorganic P; Conc.HCl-Pi, concentrated HCl-extractable inorganic P; Conc.HCl-Po, concentrated HCl-extractable organic P; Residual-P, residual inorganic and organic P; AP, available P; PAC, phosphorus activation coefficient; Ortho-P, orthophosphate; Mono-P, monoester phosphate; Diester-P, diester; Pyro-P, pyrophosphate).

The relative contribution of each P fraction to the change in TP during soil development can be determined between the change in that P faction relative to TP (Fig. S3). Soil available P (AP) had a positive correlation with NaHCO_3_-Pi, and negatively correlated with soil pH (P < 0.01) (Fig. 5). Path analysis showed that AP, Dil.HCl-Pi, Pyro-P and Conc.HCl-Po had a significantly positive effect on PAC, whereas NaOH-Po and Residual-P had significantly negative effect on this factor (Fig. 4). Additionally, TP, AP and PAC were positively correlated with easily-labile P, moderately-labile P and non-labile P (Fig. S4, *p* < 0.01).

Correlation analysis showed that Ortho-P was significantly and positively correlated with exchangeable bases, and negatively correlated with NaOH-Po, Conc.HCl-Po and SOC (P < 0.05) (Figs. 4 and 5). Mono-P had a significant positive correlation with NaOH-Po, Conc.HCl-Po and SOC, and negative relationship with Ortho-P, whereas Pyro-P and Ortho-P were significantly and positively correlated (P < 0.05). Additionally, no relationship between soil phosphatase activities and Conc.HCl-Pi and Residual-P was found in this study (P > 0.05), however, soil AcP and NeP activities had a significant positive correlation with soil PAC, AP, NaHCO_3_-Pi, NaHCO_3_-Po, NaOH-Pi, NaOH-Po, Dil.HCl-Pi and Conc.HCl-Po (P < 0.05) (Figs. 4 and 5). Positive relationships were found between AcP activity and SOC, sand and soil Mono-P (P < 0.05). Negative relationships were found between AcP activity with soil Ortho-P and clay (P < 0.05). Soil NeP activity had a significant positive correlation with sand and AcP, and was negatively correlated with pH, clay, silt and total Fe (P < 0.05). No relationship was found between soil phosphatases, Diester-P and Pyro-P (P > 0.05).

**Figure 6:**
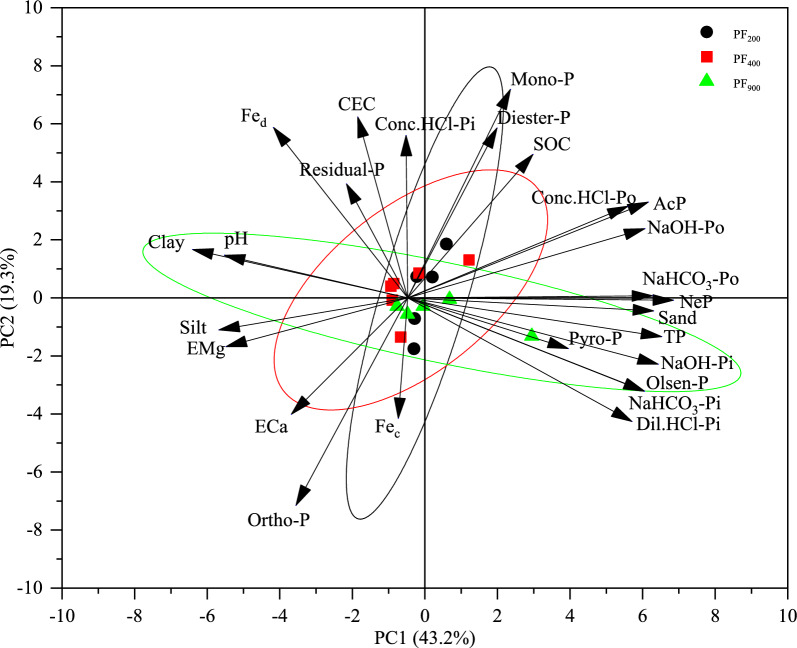
Principal component analysis (PCA) of different P species by ^31^P-NMR, sequential chemical extraction with total and available P and soil properties in paddy soils with different cultivation ages. Black, red and green solid circles represent 95% confidence for PF_200_, PF_400_ and PF_900_, respectively. (SOC, soil organic C; CEC, cation exchange capacity; ECa, exchangeable calcium; EMg, exchangeable magnesium; Fed, free iron; Fec, active iron; AcP, acid phosphatase activities; NeP, neutral phosphatase activities; Pi, inorganic P; Po, organic P; NaHCO_3_-Pi, NaHCO_3_-extractable inorganic P; NaHCO_3_-Po, NaHCO_3_-extractable organic P; NaOH-Pi, NaOH-extractable inorganic P; NaOH-Po, NaOH-extractable organic P; Dil.HCl-Pi, dilute HCl-extractable inorganic P; Conc.HCl-Pi, concentrated HCl-extractable inorganic P; Conc.HCl-Po, concentrated HCl-extractable organic P; Residual-P, residual inorganic and organic P; Olsen-P, available P; PAC, phosphorus activation coefficient; Ortho-P (AP), orthophosphate; Mono-P, monoester phosphate; Diester-P, diester; Pyro-P, pyrophosphate; PF_200_, PF_400_, and PF_900_ were the cultivation ages of 200, 400-yr and 900-yr, respectively).

**Figure 7:**
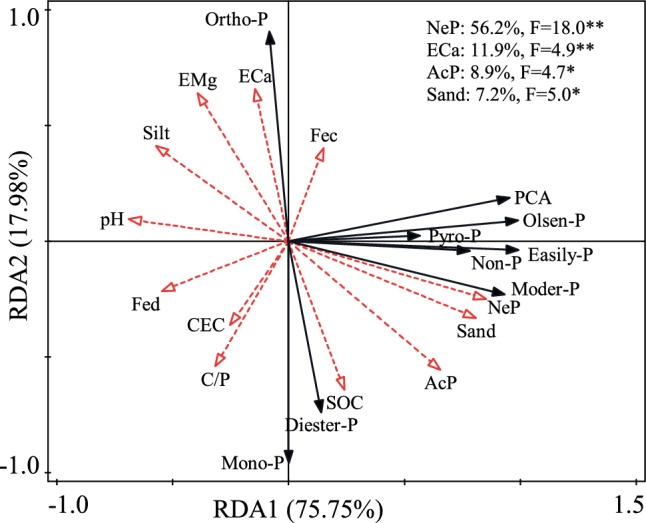
Two-dimensional sequence diagram of redundancy analysis (RDA) between soil properties and P fractions and speciation by ^31^P-NMR, sequential chemical extraction with total and available P in different paddy soils with different cultivation ages, respectively (SOC, soil organic C; CEC, cation exchange capacity; ECa, exchangeable calcium; EMg, exchangeable magnesium; Fe_d_, free iron; Fe_c_, active iron; C/P, the ratio of soil organic C and total P; AcP, acid phosphatase activities; NeP, neutral phosphatase activities; Olsen-P (AP), available P; PAC, phosphorus activation coefficient; Easily-P, easily-labile P; Moder-P, moderately-labile P; Non-P, non-labile P; Ortho-P, orthophosphate; Mono-P, monoester phosphate; Diester-P, diester; Pyro-P, pyrophosphate).

The PCA analysis of total P, available P, different P speciation and soil properties in soils with different cultivation ages is shown in Fig. 6. The two principal components axes explained 62.5% of the total variability. The dots of different cultivation age samples were mainly distributed along the horizontal axis (Fig. 6). Furthermore, it looks like there is a gradient between the three groups. The 200-yr site was about 20 degrees from vertical, the 400-yr site is about 45° from vertical, and the 900-yr site is about 100° from vertical (Fig. 6). Though the 900-yr site is in a different quadrant (Fig. 6). To confirm the degree of effect of soil properties on P fractions and species, the two-dimensional sequence diagram of redundancy analysis (RDA) was conducted (Fig. 7). The biplot space originated by the first two ordination axis of the RDA explained 93.7% of the total variance, with 75.7% rooting in the first ordination axis and 18.0% rooting in the second ordination axis (Fig. 7). The contribution rates of NeP (56.2%), exchangeable Ca (11.9%), AcP (8.9%) and sand (7.2%) on P species were significantly higher than other soil properties, thus NeP, exchangeable Ca, AcP and sand were the key factors to affect P species.

## Discussion

### Effects of cultivation age on soil P composition by sequential chemical extraction and solution ^31^P NMR

We examined the effects of long-term cultivation on P composition and cycling in paddy soils with three different ages (200, 400-yr and 900-yr). These paddy soils from PRD region had received similar fertilizer applications including chemical fertilizer for crop production. Our results showed that long-term cultivation and management in paddy soils caused marked changes in P concentrations, especially for inorganic P which ranged from 30.8 to 75.5% of TP (Fig. 1). In our study, soil total P (TP) accumulated in the topsoil and decreased with depth with the increasing of cultivation ages (Fig. 1). This increase in upper horizons results from receiving annual inputs of chemical fertilizers and/or manure. While these P sources increased production, they can also be important sources of P into surface waters due to leaching, runoff erosion^29^. Previous studies have reported the accumulation of a large amount of inorganic P (Pi) or organic P (Po) in the topsoils due to the continuous application of P fertilizer or organic manure for many years^30,31^, thus increasing the TP content. Our study had the same fertilizer application level as same as local farmers, but soil TP content increased along cultivation age increasing in the topsoil (Fig. 1). However, there were no similar changes in the other soil horizons, which could be attributed to the loss of soil P due to underground water after the application of chemical fertilization, especially P fertilizer.

Additionally, mineral P, as Ca-bound P (determined by Dil.HCl-Pi), non-labile P (Conc.HCl-Pi), and highly resistant P (occluded P, Residual-P), were detected in all types of P fractions (Fig. 1). This degree of detection was due to the high concentration of non-labile P^32^, which readily transforms between microbial biomass and non-labile P. Soil P immobilization by microorganisms sustains high P availability following microbial biomass turnover^33^. Hence, soil P is mainly associated with Ca oxides and seasonal availability is sustained from P immobilization by microbes. Higher concentrations of easily-labile Pi (NaHCO_3_-Pi), moderately-labile Po (NaOH-Po) and non-labile Pi (Conc.HCl-Pi) are found in surface (Ap1 and Ap2) horizons (Figs. 1 and 2). In contract to the 200-yr and 400-yr old soils, the G horizon in the 900 years soil has a higher concentration of these three P pools. This fact can be explained by the continuous application of fertilizers with increasing time and sustained microbial activity, maintaining some P in mobile forms that are more easily translocated through the soil^30^. Moreover, application of organic fertilizers provided readily available N to plants, which increases plant biomass. The crop residues that are generated by these applications are incorporated, resulting in accumulations of organic matters (i.e. organic C and N)^30^. For rice cultivation, soils are typically water-submerged during most of the rice growing season, resulting in the increase of water-soluble P from the hydrolysis of Fe and Al phosphates, the release of P held by anion exchange on clay and hydrous oxides of Fe and Al and the reduction of Fe^3+^ to Fe^2+^ with liberation of sorbed and chemically bonded P^11^. Moreover, the alternation of wetting and drying of rice cultivation may result in seasonal reduction and solubilization of soil Fe and Mn oxides, and contribute to the release of both adsorbed and precipitated P^34^. That may be because that P desorbed during submergence of the paddy soils has migrated and sorbed to iron oxides and clays in the subsoils^11^. Moreover, paddy soils are derived from a range of parent materials with different sensitivities to anthropogenic activities and likely different pedogenic thresholds for nutrient retention. Therefore, long-term sustainability of paddy cultivation affects the forms of Pi and Po and their transformation due to solubilization of soil Fe and Mn oxides and the changes of soil clay and organic matters. Huang et al.^11^ deemed that anthropogenic activities (i.e. paddy cultivation) alter both the rate and trajectory of P transformations during the early stage of paddy soil development. Additionally, our results show translocation of P has occurred into the subsoil and demonstrate the conversion between Po and Pi forms and their respective conversion into soluble P due to soil microbial activity and fertilization.

The analysis with ^31^P NMR showed that P composition was influenced by cultivation age and soil depth (Table 3, Fig. 2). Ortho-P was the major extractable Pi speciation in all paddy soils and increased with time of cultivation (Table 3, Fig. 2). Mono-P dominated organic P forms as they are stabilized in soils by association with amorphous metal oxides and can also be produced by alkaline degradation of RNA and phospholipids^35^. The increasing inorganic P (including Pyro-P) with increasing cultivation age (Table 3, Fig. 2) could be attributed to the increased mineralization under long-term cultivation and tillage^35^. Furthermore, ^31^P NMR showed that organic P mainly consisted of Mono-P and Diester-P, which was in agreement with other studies^36^. Howerer, Diester-P is more readily degraded by microbes and enzymes than Mono-P^37^. Thus, the depletion of Pi results in a greater reliance on Po cycling via mineralization, i.e., depletion of the normally recalcitrant Diester-P pool^37^.

Likewise, the relatively large fraction of total organic P extracted with HCl, which is not available for plant uptake, is a very important P reserve^30^. This will help buffer P availability via mineralization of organic P by phosphatases^38^, once labile and solution P are insufficient for plant growth^39^. In addition, under the condition of dry–wet cycling of paddy soils, solution P can readily form stable compounds with iron, while organic P can be translocated deeper into the soil^40^. Overall, long-term anthropogenic cultivation affects soil P composition, P activation and transformation.

### Effects of phosphatase activities and soil properties on P compositions

Soil enzymes are important indicators of biological property changes of soils because of their sensitivity to soil management practices^13^. In this study, soil AcP and NeP activities were highest in the topsoil and decreased with soil depth (Fig. 3). Both enzymes increased in topsoils over time from both addition of amendments and long-term agricultural cultivation (Fig. 3). This increase occurs as soil phosphatase activity is significantly influenced by soil microbial activity^30^. Long-term agricultural cultivation and fertilizers can enhance soil microbial activities^39^. Microbial activity would be increased with increasing C sources resulting from residue inputs^13^. Enzyme activities increase with increasing residue inputs from long-term paddy cultivation (Fig. 3). Therefore, the increasing activity could be attributed to two factors: (1) increased substrates of soil phosphatases from crop residues and (2) increased microbial activities as soil phosphatases are believed to be derived primarily from microorganisms^13,41^.

The AcP activity was significantly and positively correlated with soil AP, SOC, TP and sand (Fig. 4). These positive correlations demonstrate that AcP is a sensitive index for nutrients storage and could be used as an indicator of soil quality^42^. In addition, the NeP activity was significantly and positively correlated with TP and AP, suggesting that NeP might play an important role in the hydrolysis and mobilization of soil-bound P in these soils^42^. Also, AcP had a negative relationship with Ortho-P, but was positively correlated with Mono-P, suggesting that Ortho-P and Mono-P were influenced and regulated by AcP activity (Fig. 4). Significant positive correlations were found among AcP, NeP and easily-labile P (NaHCO_3_-Pi and NaHCO_3_-Po), moderately-labile P (NaOH-Pi and NaOH-Po) and non-labile P (Conc.HCl-Po and Dil.HCl-Pi) (Fig. 4), indicating that AcP and NeP likely play an important role in transformation of soil P compositions in these soils^42^.

Additionally, soil phosphatases catalyze the hydrolysis of organic compounds containing P, releasing inorganic P and thus increasing soil available P^43^. In fact, different phosphatases have unique effects on individual P composition^41^. Briefly, monoesters can be hydrolyzed by AcP and NeP to release Ortho-P. High inorganic P concentrations may reduce phosphatase activities in long-term cultivated soils by inhibiting secretion of soil phosphatase by microorganisms and plants^44^. Also, organic phosphate compounds are not readily degraded by soil microorganisms and phosphatases in acid soils due to the stronger adsorption capability of low pH soils^45^.

Correlation analysis showed that soil pH was significantly correlated with Pi, while SOC was significantly correlated with Po (Fig. 5). This correlation indicates that the availability of P is impacted by both pH value and SOC concentration. Although parent material and soil pH have considerable influence on the distribution of inorganic phosphates between Ca (P_Ca_) and metal oxide (Fe and Al) sorbents (P_Fe+Al_)^30,32^, the three soils were developed from similar alluvial parent materials. Thus, we quantitatively assessed the Pi fraction between these sorbents by assigning acid-extractable P (Dil.HCl-Pi) to Ca sorbents and base-extractable P (NaHCO_3_-Pi + NaOH-Pi) to metal oxide sorbents and compared the P to Total-P proportion in each of these extracts. The soils at all three sites, with pH maintained between 6.2 and 7.0, released 1 to 4 times P contents in the acid extraction than in the alkaline extraction (Table 1, Fig. S2), consistent with a larger amount of P associated with inorganic Ca minerals than that with Fe and Al oxides^15^. Thus, long-term paddy cultivation may be a soil-degrading pathway as the loss of soil materials (e.g., Fe, Al oxides, pH and SOC) by physical and chemical processes damage the P fractions and compositions (and possible other nutrient) and P holding capacity.

### Soil P availability influenced by cultivation age and soil depth

Soil available P (AP) is considered a major factor in both evaluating the soil P supply capacity and determining the P fertilization rate. Moreover, Olsen-P was referred to as ‘available P’ to emphasize its availability in this study. Soil AP in highly weathered soils is generally low and depends on organic phosphate mineralization^39^. Soil Po in the available pool (NaHCO_3_-Po) is very important because it increases the apparent P availability^46^. Similarly, Po that occurs in the moderately-liable (NaOH-Po) pool is as important as NaHCO_3_-Po because it contributes to P reserves^39^. TP represents the long-term potential of the P supply, whereas easily-labile Pi represents the short-term bioavailability^2^. Easily labile P, moderately liable P and non-available P increased in surface horizons (Ap1 and Ap2) with increasing cultivation age (Fig. 2). Phosphorus in these pools is contributing to the increase of total P in long-term paddy cultivation through fertilization (Fig. 1). Furthermore, easily liable P, moderately liable P and non-available P in the Br2 and G horizons declined with cultivation age between 400 and 900 years, whereas these soils had high P contents at Ap1 and Ap2 horizons. These trends indicate that P could accumulate at tropsoil due to paddy cultivation and P fertilizer^47^.

In this study, soil Dil.HCl-Pi and Pyro-P increased with increasing cultivation age (Table 3, Figs. 1 and 2), which indicated that long-term paddy cultivation increased the pool of soil inorganic P. This is probably due to microbial decomposition and mineralization of organic soil amendments from cultivation, with more secondary phosphate minerals formed resulting in more inorganic P in topsoils^30,39^.

In many ecosystems, the pool of P is considered the regulator for soil C and N cycles^48^. Furthermore, P is an essential macronutrient, and its availability directly governs global crop production^2,49^. Our results extend investigations of decadal changes in surficial P speciation in agricultural systems to millennial scales of profile changes, indicating that long-term agricultural cultivation may be an extremely important factors affecting the transformation of soil P forms in the profile. Therefore, characterizing the distribution and transformation characteristics of soil P fractions and compositions by various methods is of great significance in order to understand biogeochemical cycling of P pool in terrestrial ecosystem.

## Conclusions

This study examined the changing of P fractions with three paddy soils of different cultivation ages using sequential fractionation and ^31^P Nuclear Magnetic Resonance methods. Distributions of soil inorganic and organic P fractions were strongly influenced by cultivation ages. Soil inorganic P contents were higher than organic P at all three paddy soils. Organic P was primarily composed of HCl-Po and NaOH-Po, with NaHCO_3_-Po comprising a small fraction, indicating that availability of total organic P was low especially in subsoils. Moreover, acid phosphatase enzyme had higher activity than neutral phosphatase in these soils, which was significantly positive correlation with phosphate monoesters and negative correlation with orthophosphate. Long-term cultivation appeared to improve soil phosphatase activities, and increased concentrations of orthophosphate and decreased phosphate monoesters and diesters indirectly, further promoted the transformation of organic P to inorganic P. Soil organic P mineralized to inorganic P in long-term agricultural cultivation plays a vital role to the increase of soil P availability. From these perspectives, understanding the factors driving P cycling and biological P availability is critical for maximizing agricultural productivity and proper use of applied nutrients under long-term agricultural cultivation.

## Methods

### Site description and cultivation age recognition

The Pearl River Delta (PRD) (21°34′–23°45′ N, 112°07′–114°39′ E), an area within Guangdong Province of south China, characterized as a subtropical monsoon climate zone with a mean annual temperature of 21–24 °C and mean annual precipitation of 1301–2700 mm. In this region, Jiangmen city and Fuoshan city were selected to sample (Table S1). Jiangmen city (21°27′–22°51′ N, 111°59′–113°15′ E) is located on the west bank of the Pearl River Delta, which belongs to Guangdong Province of south China and has a mean annual air temperature of 22.0 °C and a mean annual precipitation of 2078 mm. Foshan (23°02′ N, 113°06′ E) city is located on the back-land of the Pearl River Delta, which belongs to Guangdong Province of south China and has a mean annual air temperature of 23.2 °C and a mean annual precipitation of 1653 mm. Jia et al.^50^ collected a series of Jiangmen City and Fuoshan City annals that record in detail the rice cultivation situations according to the history of reclamation in the PRD region from zhao and Yang^19^ and Deng^51^. This chronology was used in the current study to estimate the ages of paddy cultivation^11^. Thus, the cultivation ages in the paddy soils of the PRD region were obtained by Jia et al.^50^ including three profiles with the cultivation ages of 200 (PF_200_), 400-yr (PF_400_) and 900-yr (PF_900_) (Table S1). According to the World Reference Base for Soil Resources^52^, the soils in the paddy field were classified as Stagnic Anthrosols derived from river alluvium parent material. All three sites were on geomorphically stable topographic positions without slope, relatively consistent climatic conditions and same parent material, minimizing the effect of topographic, climatic and parent material.

In the PRD region, paddy cultivation management is nearly identical within each chronosequence, as confirmed by local farmers and our field observations. The dominant rice cropping systems at Jiangmen and foshan cities are all double rice, in which usual P fertilizer application rates were all approximately 40–80 kg P_2_O_5_ ha^−1^ yr^−1^ in the form of inorganic calcium superphosphate at Jiangmen and Foshan cities. Moreover, N and K fertilizer application rates were approximately 130 ~ 200 kg ha^−1^ yr^−1^ of urea (46% N) and 50 ~ 100 kg ha^−1^ yr^−1^ of potassium chloride (60% K_2_O). This estimation is based on extensive surveys with local farmers and governmental agricultural advisors in the PRD region. In addition, all rice straws were returned to the paddy field every year according to local farmers. Thus, P removal by crops was balanced by P input from anthropogenic fertilization (e.g., human and livestock manure and crop straws) to maintain stable crop yields since there is no record of declining yields^19^.

Within each area of identical paddy cultivation history, two representative paddy soil profile sites (PF_200_ and PF_400_) were chosen in jiangmen city and one representative paddy soil profile site (PF_900_) were chosen in Foshan city for soil sampling based on soil landscape and geomorphological characteristics of the PRD area. We selected three sampling plots (each plot was 5.0 × 5.0 m^2^) in the three paddy soil sites when the fields were drained after rice harvest in November 2015 (Table S1). Then, three subplots with a size of 1.0 × 1.0 m^2^ for paddy soils, which were selected randomly at each plot, were dug to collect one soil samples at each genetic horizon (Table S1). Soil profiles were described and sampled according to genetic horizons following standard field description guidelines by Zhang and Li^53^. A total of 16 samples were collected from three representative paddy soils with different cultivation ages (Table S1). All samples were divided into two parts: one sample air-dried indoors for analysis of soil parameters and P fractionation; and another sample kept field moist and refrigerated at 4 °C for enzyme analysis.

### Analysis of soil parameters and sequential P fractionation

Air-dried soils were sieved sieved to 2-mm and 0.15-mm prior to analysis, respectively. Characterization methods used are documented in Zhang et al.^54^. Soil pH was determined using a pH electrode (PB-10, Beijing Selidos Scientific Instruments Co, LTD) in distilled water (1:2.5 soil:solution ratio). Particle size analysis determined sand (2 to 0.05 mm), silt (0.05 to 0.002 mm), and clay (< 0.002 mm) using the pipette method following hydrogen peroxide treatment. Soil organic carbon (SOC) was measured using a heated dichromate oxidation method. Cation exchange capacity (CEC) was determined with 1.0 M ammonium acetate (pH 7.0). Exchangeable Ca^2+^ (ECa) and Mg^2+^ (EMg) was measured using a boric acid-atomic absorption spectrometry method. Active iron (Fe_c_) was determined with ammonium oxalate extraction and free iron (Fe_d_) with dithionite-citrate-bicarbonate (Table 1). Total P was determined by two methods: (1) TP was determined by HNO_3_-HF-HClO_4_ digestion followed by molybdenum antimony anti-colorimetric analysis on a ultraviolet spectrophotometer; (2) TP_sum_ was obtained by the sum of inorganic P and organic P fractions by sequential extraction method. Available P (AP or Olsen-P) was determined by extracting soils with sodium bicarbonate at pH 8.5 using molybdenum antimony anti-colorimetric analysis on a ultraviolet spectrophotometer (Table 2).

We used the Hedley et al.^55^ sequential extraction procedure as modified by Tiessen and Moir^56^ to assess soil P fractions (Table S2). Samples were sequentially extracted using 0.5 M NaHCO_3_, 0.1 M NaOH, 1.0 M HCl, and hot concentrated HCl, and a final digestion of the residue with HNO_3_-HF-HClO_4_ using colormetric method^3^. In order to facilitate interpretations, P fractions were classified into three main groups^56^: (i) easily-labile P, P that is considered readily available for plants, i.e., NaHCO_3_-Pi and NaHCO3-Po; (ii) moderately-labile P, P that is strongly adsorbed by Al and Fe oxides, i.e. NaOH-Pi and NaOH-Po; (iii) non-labile, that P associated with calcium (Ca-P) and Residual-P, considered as non-available P to plants, i.e. HCl-P and Residual-P. In this study, the organic matter contents of soil samples were 0.7–3.9% (Table 1), thus we classified Residual-P as inorganic P due to the lower organic matter contents^7^. Inorganic P (Pi) is the sum of P fractions with NaHCO_3_-Pi, NaOH-Pi, Dil.HCl-Pi, Conc.HCl-Pi and Residual-P, while organic phosphorus (Po) is the sum of all fractions of NaHCO_3_-Po, NaOH-Po and Conc.HCl-Po, respectively.

The accuracy of the TP by HNO_3_-HF-HClO_4_ digestion was assessed by comparing with the sum of all the Pi and Po fractions (TP_sum_), with an expected 1:1 relation between the two parameters for all soil horizons (Fig. S1, n = 15). The null hypothesis of a linear relation could not be disproved (Fig. S1). The two measures were very strongly correlated (r^2^ = 0.98). Thus, the efficiency of sequential extraction for the soils in this study was excellent.

### ^31^P nuclear magnetic resonance analysis

A modified procedure of Cade-Menun and Preston^57^ was used for solution ^31^P NMR spectroscopy to identify P species including Pi (orthophosphate, Ortho-P; pyrophosphate, Pyro-P) and Po (orthophosphate monoesters, Mono-P; diesters, Diester-P). Ground samples (3 g) were shaken with 60 mL of 0.25 M NaOH and 0.05 M Na_2_EDTA in a 100-mL centrifuge tube at 20 °C for 16 h. The extract was centrifuged (10,000×*g* for 30 min at 16 °C), and the supernatant filtered with a 0.45 μm membrane, and then 15 mL of the filtrate was freeze dried (lyophilized). The powder was dissolved in 1 mL of 0.25 M NaOH, and centrifuged at 8000×*g* for 5 min at 4 °C. Then, 0.6 mL of the supernatant was removed into a 5-mm NMR tube, followed by 0.05 mL of deuterium oxide (D_2_O) for signal locking. Meanwhile, 5 mL of the filtrate was subjected to digestion (H_2_SO_4_-HClO_4_) and measured to determine total P (TP_alkali_).

Solution ^31^P NMR spectra were obtained using a Bruker AMX 600 spectrometer (Bruker, Germany) operating at 243 MHz with a 5-mm probe^58,59^. We used a 30° pulse width, a total acquisition time of 1.5 s (pulse delay 0.808 s, acquisition time 0.673 s) and broadband proton decoupling (additional spectra were acquired for some samples without proton decoupling). The delay time used here allows sufficient spinlattice relaxation between scans for P compounds in NaOH-EDTA, confirmed by a recent detailed study of relaxation times for P compounds in various soil extractants^60^. Temperature was regulated at 24 °C. Between 80 and 400 scans were collected to obtain acceptable signals. Chemical shifts were measured relative to an external standard of 85% H_3_PO_4_ after Lorentzian convolution with a width of 10 Hz. We used the deconvolution process of the Bruker WinNMR program to determine chemical shift, line width, and area of individual signals. Spectra were plotted using a line broadening of 5 Hz. Spectra were collected immediately after preparation (usually within 1 h) and repeated at intervals during storage at room temperature to determine degradation, with the same number of scans collected each time. No degradation was observed unless specified^58,61^.

### Phosphatase enzyme hydrolysis assay

Moist soil samples were extracted in duplicate with distilled water (1:4 soil-solution ratio) on a reciprocating shaker for 1 h^62^. The extract was centrifuged at 10,000×*g* for 10 min and filtered through 0.45-mm membrane filters. Two phosphatase enzyme activities involved in P cycles were determined. Acid (AcP) and neutral (NeP) phosphatase activities were assayed with p-nitrophenol phosphate as substrates with modified universal buffer pH values of 5 and 7, respectively. The activities of AcP and NeP were expressed as mg p-nitrophenol kg^−1^ soil (dry weight) h^−1^.

### Data analysis

Phosphorus activation coefficient (PAC) is an important indicator of soil P availability and the transformation of P fractions^63^. The higher PAC means more strongly activation ability of soil P, thus promotes the uptake of P by plants. Soil PAC is related to AP and TP as follows:

1 $${\text{PAC }}\left( \% \right) \, = {\text{ AP }}\left( {{\text{mg kg}}^{{ - {1}}} } \right) \times {1}00/[{\text{TP}}\left( {{\text{g kg}}^{{ - {1}}} } \right) \times {1}000]$$

Microsoft Excel 2016 and Origin 2021b (OriginLab, New York, USA) were used for data processing and cartography. Data were statistically analyzed using IBM SPSS Statistics 24.0 (SPSS Inc. Chicago, USA) software. Pearson correlation analyses were used to identify relationships between different P species and total P, available P contents and soil physicochemical properties. Principal Component Analysis (PCA) was used to evaluate the overall differences among P species and the soil properties in paddy soils.

Nuclear magnetic resonance data were processed using the Mnova 5.3.1 (Qingdao Tenglong Microwave Technology Co, LTD). Concentration of P species in soil samples identified in ^31^P NMR spectrum were calculated using the corresponding peak area^64^ and ^31^P NMR spectra were plotted with Origin 2021b. Proportional soil concentration of specific P species identified in the ^31^PNMR spectra were calculated using the corresponding peak area and the recovery of P in NaOH-EDTA extracts relative to the corresponding soil P contents^64^. By spectrum analysis, Ortho-P and Pyro-P were averaging at 6.0 ppm and 4.0 ppm chemical shift, respectively, and the chemical shifts of Mono-P and Diester-P were between 3.0–5.0 ppm and − 2.0–0.0 ppm (Table 3, Fig. 2).

Relationship between selected soil properties and P compounds were evaluated with correlation, linear regression and path analyses using SPSS 24.0 (IBM, SPSS, New York, USA). Correlation heat map and PCA were used to reduce the number of variables and understand the relationships between selected soil properties and P compositions using Origin 2021b. Redundancy analysis (RDA) was used to visualize the relationships between response variables (i.e., P compositions) and explanatory factors (i.e., soil properties) using Canoco 5.0 (Canoco, NY, USA).

## Supplementary Information

Supplementary Information.

## Data Availability

All the data generated or analyzed during this research are included in this published article and its supplementary information flies. The datasets used and/or analyzed during the current study available from the corresponding author on reasonable request.
