# Supplementary material for: Organic/inorganic phosphorus partition and transformation in long-term paddy cultivation in the Pearl River Delta, China
Source: Sci Rep. 2023 Jul 10;13:11122. doi: 10.1038/s41598-023-38369-2 (PMC10333323; doi:10.1038/s41598-023-38369-2)
Supplement: Supplementary file 1 — Supplementary Information. [file 41598_2023_38369_MOESM1_ESM.docx]

### **Table S1** Basic information of the soil sampling sites.

| Sampling NO. | Soil genetichorizon | Depth  (cm) | Sampling site | Parent materials | Land uses | Soil type | Cultivation age (a) | Altitude (m) |
| --- | --- | --- | --- | --- | --- | --- | --- | --- |
| PF_200_ | Ap1 | 0-14 | Xinhui district, Jiangmen city, Guangdong province | River alluvium | Paddy | Hydragric Anthrosols | 200 | 20 |
|  | Ap2 | 14-23 |  |  |  |  |  |  |
|  | Br1 | 23-60 |  |  |  |  |  |  |
|  | Br2 | 60-90 |  |  |  |  |  |  |
|  | G | 90-120 |  |  |  |  |  |  |
| PF_400_ | Ap1 | 0-13 | Xinhui district, Jiangmen city, Guangdong province | River alluvium | Paddy | Hydragric Anthrosols | 400 | 20 |
|  | Ap2 | 13-28 |  |  |  |  |  |  |
|  | Br1 | 28-45 |  |  |  |  |  |  |
|  | Br2 | 45-73 |  |  |  |  |  |  |
|  | Br3 | 73-92 |  |  |  |  |  |  |
|  | G | 92-118 |  |  |  |  |  |  |
| PF_900_ | Ap1 | 0-19 | Nanhai district, Fushan city, Guangdong province | River alluvium | Paddy | Hydragric Anthrosols | 900 | 20 |
|  | Ap2 | 19-32 |  |  |  |  |  |  |
|  | Br1 | 32-59 |  |  |  |  |  |  |
|  | Br2 | 59-82 |  |  |  |  |  |  |
|  | G | 82-113 |  |  |  |  |  |  |

PF_200_, PF_400_, and PF_900_ were the cultivation ages of 200, 400-yr and 900-yr, respectively.

### Table S2 Extracting reagents for each fraction of soil P extracted in sequential fractionation procedure.

| Extractant | Supernatant treatment | P fraction extracted |
| --- | --- | --- |
| (1) 0.5-g soil  30 mL 0.5 M NaHCO3† pH 8.5, shake for 16 hours | NaHCO_3_-Pi and NaHCO_3_-Po, determine total P (TP). Precipitate organic matter, determine Pi | Labile P: Pi weakly associated with Fe and Al at mineral surfaces; Po associated with soil organic surfaces and humic and fluvic acids. Regarded as the plant available pool (Tiessen and Moir, 1993) |
| (2) 30 mL 0.1 M NaOH, shake for 16 hours | NaOH-Pi and NaOH-Po, determine total P (TP). Precipitate organic matter, determine Pi | Pi more strongly associated with Fe and Al at mineral surfaces. Po more strongly associated with soil organic surfaces and humic and fluvic acids. Intermediate P lability pool (Tiessen and Moir, 1993) |
| (3) 30 mL 1.0 M HCl, shake 16 hours | Dil.HCl-Pi, determine Pi | Ca-associated Pi (P_Ca_), weatherable mineral |
| (4) Hot conc. HCl (80℃) | Conc.HCl-Pi and Conc.HCl-Po, determine TP and Pi directly | Stable occluded Pi released from the dissolution of sesquioxides. Stable Po and Po from particulate organic matter that is not alkali extractable, but may be bio-available (Tiessen and Moir, 1993) |
| (5) HClO_4_-HF | Residual-P, determine TP and Pi directly | Recalcitrant residue Pi |

† This bicarbonate fraction is not comparable to the widely used Olsen test for P (Olsen et al, 1954) because the resin will have already removed P, which would have been extracted by the Olsen method. (NaHCO_3_-Pi, NaHCO_3_-extractable inorganic P; NaHCO_3_-Po, NaHCO_3_-extractable organic P; NaOH-Pi, NaOH-extractable inorganic P; NaOH-Po, NaOH-extractable organic P; Dil.HCl-Pi, dilute HCl-extractable inorganic P; Conc.HCl-Pi, concentrated HCl-extractable inorganic P; Conc.HCl-Po, concentrated HCl-extractable organic P; Residual-P, residual inorganic and organic P).





### **Fig. S1** Relationship between the sum of all the P fractions (TP_sum_) and total soil P (TP) measured by HClO_4_-HF digestion.

**Fig. S2** Distribution of easily-labile, moderately-labile and non-labile soil P in soil profiles across three soil cultivation ages. The depth of a specific soil horizon is not directly comparable to the width of bar. (Easily-labile P, the sum of NaHCO_3_-Pi and NaHCO_3_-Po; Moderately-labile P, the sum of NaOH-Pi and NaOH-Po; Non-labile P, the sum of Conc.HCl-Po, Conc.HCl-Pi, Residual-P and Dil.HCl-Pi; NaHCO_3_-Pi, NaHCO_3_-extractable inorganic P; NaHCO_3_-Po, NaHCO_3_-extractable organic P; NaOH-Pi, NaOH-extractable inorganic P; NaOH-Po, NaOH-extractable organic P; Conc.HCl-Po, concentrated HCl-extractable organic P; Residual-P, residual inorganic and organic P; Conc.HCl-Pi, concentrated HCl-extractable inorganic P; Dil.HCl-Pi, dilute HCl-extractable inorganic P; PF_200_, PF_400_, and PF_900_ were the cultivation ages of 200, 400 years and 900 years, respectively).

### **Fig. S****3** Changes in soil P pools (mg kg^-1^) at different soil depths of each cultivation age. Pi, inorganic P; Po, organic P. NaHCO3-Pi, NaHCO3-extractable inorganic P; NaHCO3-Po, NaHCO3-extractable organic P; NaOH-Pi, NaOH-extractable inorganic P; NaOH-Po, NaOH-extractable organic P; Dil.HCl-Pi, dilute HCl-extractable inorganic P; Conc.HCl-Pi, concentrated HCl-extractable inorganic P; Conc.HCl-Po, concentrated HCl-extractable organic P; Residual-P, residual inorganic and organic P; PF_200_, PF_400_, and PF_900_ were the cultivation ages of 200, 400-yr and 900-yr, respectively; PF_400_ vs. PF_200_, the difference value between PF_400_ and PF_200_; PF_900_ vs. PF_400_, the difference value between PF_900_ and PF_400_.


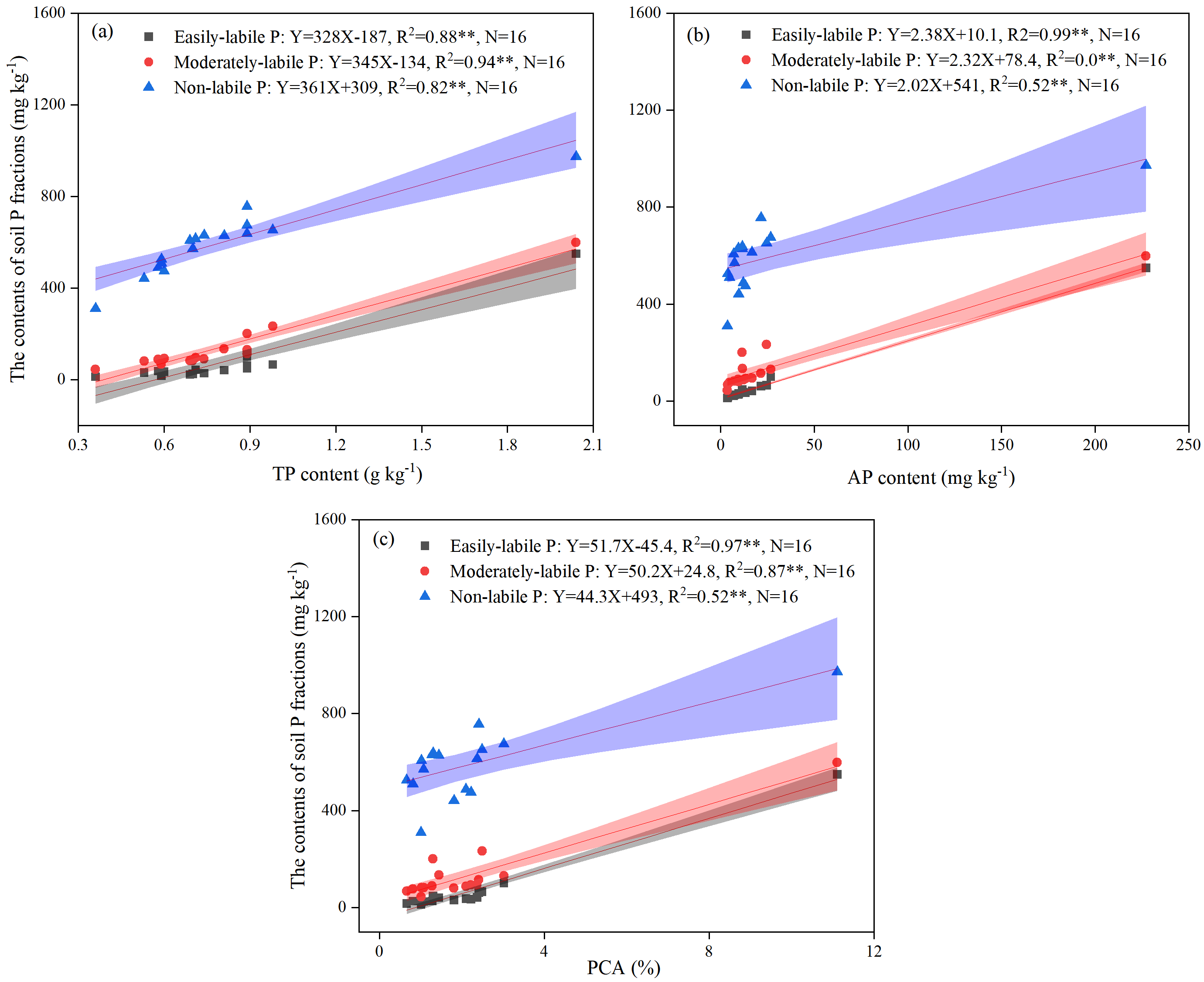


### **Fig. S4** Linear correlation relation among soil P fractions, total P (TP, a), available P (AP, b) and P activation coefficient (PCA, c). (Easily-labile P, the sum of NaHCO_3_-Pi and NaHCO_3_-Po; Moderately-labile P, the sum of NaOH-Pi and NaOH-Po; Non-labile P, the sum of Conc.HCl-Po, Conc.HCl-Pi, Residual-P and Dil.HCl-Pi).
